# Good practices for Ga-68 radiopharmaceutical production: an important correction

**DOI:** 10.1186/s41181-022-00184-x

**Published:** 2023-01-24

**Authors:** Aziz Gültekin

**Affiliations:** Department of Nuclear Medicine, Pamukkale University, Denizli, Turkey

## To the editor

The review titled “Good practices for ^68^Ga radiopharmaceutical production” by Nelson et al. (2022) and published in the latest issue of your journal contains important information about ^68^Ga radiopharmaceuticals. However, sentence of the “These ^68^Ga radiopharmaceuticals include agents such as [^68^Ga]Ga-macroaggregated albumin for myocardial perfusion evaluation…” contradicts basic nuclear medicine knowledges.

^68^Ga-macroaggregated albumin is stated to be a potential radiopharmaceutical in lung perfusion studies in preclinical and clinical studies (Gültekin et al. 2020; Ament et al. 2013). ^68^Ga-macroaggregated albumin is not used in myocardial perfusion studies. I think that this error maybe caused by inattention of the author and in the review processes, and I would like to point out that it would be useful to correct it.

## Data Availability

Not applicable.
